# Factors associated with successful electrical cardioversion for atrial fibrillation

**DOI:** 10.1002/joa3.70124

**Published:** 2025-06-30

**Authors:** Naoya Kataoka, Teruhiko Imamura

**Affiliations:** ^1^ Second Department of Internal Medicine University of Toyama Toyama Japan

To The Editor,

Electrical cardioversion is occasionally employed in routine clinical practice for the management of atrial fibrillation (AF). Its success is associated with the avoidance of hospitalization. The authors have identified several predictors of AF recurrence following electrical cardioversion, such as nonhigh‐density lipoprotein cholesterol (non‐HDL C) and the number of cardioversions^1^; however, several important considerations warrant discussion.

Given the retrospective nature of the study, the scenario of electrical cardioversion likely varied across patients. For example, clinicians may have administered antiarrhythmic agents prior to performing electrical cardioversion, particularly in symptomatic individuals. Such pharmacologic pre‐treatment could increase the likelihood of achieving and maintaining sinus rhythm.^2^

Definitions of successful cardioversion differ across studies. In investigations examining the role of pre‐ablation electrical cardioversion, success has been defined as the absence of AF recurrence within 24 hours.^3^ In contrast, this study reports that 81 patients remained free from AF recurrence over a median follow‐up period of 60 months—a markedly favorable outcome that surpasses that of contemporary catheter ablation strategies.^1^

Recent literature has introduced the concept of “acute atrial fibrillation,” which arises secondary to acute systemic illnesses.^4^ In these cases, AF may resolve entirely without recurrence following elimination of the precipitating factor. It is conceivable that patients with this self‐limited form of AF were included among those deemed successfully treated in this study.

The authors propose an association between non‐HDL‐C, chronic inflammation, and AF recurrence.^1^ However, the specific mechanistic link between non‐HDL‐C levels and myocardial inflammation remains unclear. Previous studies have demonstrated a relationship between AF and chronic inflammation or chronic myocardial injury, as indicated by elevated serum levels of high‐sensitivity C‐reactive protein or troponin.^5^, ^6^ Typically, elevated non‐HDL‐C is associated with coronary artery disease, which itself may confound the observed relationship with recurrent AF. Analyzing the correlation between non‐HDL‐C levels and serum inflammatory markers or troponin may clarify the relationship among these parameters.

## FUNDING INFORMATION

None.

## CONFLICT OF INTEREST STATEMENT

The authors declare no conflict of interest.

## Data Availability

The manuscript does not include any original data.
